# Proteomic Analysis of Dendritic Filopodia-Rich Fraction Isolated by Telencephalin and Vitronectin Interaction

**DOI:** 10.3389/fnsyn.2018.00027

**Published:** 2018-08-10

**Authors:** Yutaka Furutani, Yoshihiro Yoshihara

**Affiliations:** ^1^Laboratory for Neurobiology of Synapse, RIKEN Brain Science Institute, Saitama, Japan; ^2^Laboratory for Systems Molecular Ethology, RIKEN Center for Brain Science, Saitama, Japan

**Keywords:** telencephalin, ICAM-5, vitronectin, proteomics, dendritic filopodia

## Abstract

Dendritic filopodia are thin, long, and highly mobile protrusions functioning as spine precursors. By contrast with a wealth of knowledge on molecular profiles in spines, little is known about structural and functional proteins present in dendritic filopodia. To reveal the molecular constituents of dendritic filopodia, we developed a new method for biochemical preparation of proteins enriched in dendritic filopodia, by taking advantage of specific and strong binding between a dendritic filopodial membrane protein, telencephalin, and its extracellular matrix ligand, vitronectin. When vitronectin-coated magnetic microbeads were added onto cultured hippocampal neurons, phagocytic cup-like membrane protrusions were formed on dendrites through the binding to telencephalin. Magnetically purified membrane protrusion fraction was subjected to comprehensive mass spectrometric analysis and 319 proteins were identified, many of which were confirmed to be localized to dendritic filopodia. Thus, this study provides a useful resource for studying molecular mechanisms underlying dendritic development, synapse formation, and plasticity.

## Introduction

Neuronal dendrites are equipped with two morphologically and functionally distinct types of tiny protrusions: filopodia and spines. Dendritic filopodia are long, thin and highly dynamic protrusions mainly observed in developing neurons. They continue elongation and retraction flexibly as if to search for appropriate presynaptic partners (Ziv and Smith, 1996; Fiala et al., 1998; Portera-Cailliau et al., 2003). Upon making a contact with an appropriate axon, dendritic filopodia is transformed and stabilized into a spine. Thus, dendritic filopodia is an important neuronal compartment functioning as a spine precursor. Also in adult brain, plastic changes of synapses are frequently associated with emergence of dendritic filopodia (Zuo et al., 2005; Pan and Gan, 2008; Yoshihara et al., 2009). Furthermore, morphological abnormalities of dendritic protrusions are often observed in patients’ brains with mental disorders such as autism spectrum disorders, schizophrenia, Alzheimer’s disease, Down syndrome, and Rett syndrome (Kaufmann and Moser, 2000; Penzes et al., 2011). A number of causal candidate genes responsible for these disorders have been identified and many of them turned out to have defined roles in spine and synapse development. Dysfunction of these molecules sometimes leads to abnormal dendritic morphology with less spines and more dendritic filopodia even in adulthood (Penzes et al., 2011).

In the last decade, many researchers successfully uncovered molecular organization of dendritic spines by a combinatorial approach with biochemical purification of the postsynaptic density fraction and mass spectrometry-based comprehensive proteomic analysis (Walikonis et al., 2000; Satoh et al., 2002; Peng et al., 2004; Bayes et al., 2011; Bayes et al., 2012). Thus we currently have a wealth of knowledge on structural and signaling proteins enriched in spines that play pivotal roles in synapse formation and plasticity (Benson et al., 1998). In striking contrast, molecular constituents of dendritic filopodia remain largely unknown, except for a few molecules such as a telencephalon-specific cell adhesion molecule TLCN (ICAM-5) and phosphorylated ERM family actin-binding proteins (Matsuno et al., 2006; Furutani et al., 2007). So far, there has been no report on proteomics analysis of dendritic filopodia, because of the lack of an efficient purification method for filopodia-enriched fraction.

In this study, by taking advantage of specific and strong binding between TLCN and its extracellular ligand, VN, we developed a unique biochemical method for enrichment of functional molecules present in dendritic filopodia. Proteomics analysis of the purified fraction identified 319 proteins, many of which were abundantly localized to dendritic filopodia.

## Materials and Methods

### Antibodies

Anti-TLCN-C (Sakurai et al., 1998), Anti-TLCN/Fc (Mitsui et al., 2007), anti-vitronectin (Furutani et al., 2012), anti-actin (1:1000, A-5060, Sigma-Aldrich), anti-α-actinin (1:100, A-5044, Sigma-Aldrich), anti-BAIAP2L1 (1:100, GTX109453S, GeneTex), anti-CaMKIIα (1:1000, MAB8699, Chemicon), anti-CD98 (1:200, sc7094, Santacruz), anti-eEF1γ (1:1000, NB100-2262, Novus Biologicals), anti-EPS8L1 (1:100, AV42491, Sigma-Aldrich), anti-EFA6C (1:100, 17404-1-AP, ProteinTech Group), anti-EFA6D (1:100, ab36165, Abcam), anti-Gαo (1:100, Santacruz), anti-Gαq (1:200, sc-393, Santacruz), anti-Gβ2 (1:100, ab81272, Abcam), anti-JIP4 (1:50, NB110-82383, Novus Biologicals), anti-MAP1S (1:100, 15695-1-AP, ProteinTech Group), anti-MRCKα (1:100, ab38356, Abcam), anti-myosin VA (1:100, #3402, Cell Signaling), Na^+^/K^+^ ATPase α3 (1:1000, MA3-915, Thermo Scientific), anti-NR3A,B (1:100, GTX22639, GeneTex), anti-PLCβ3 (1:200, sc-403, Santacruz), anti-PSD95 (1:1000, MA1-046, ABR), anti-ribosomal protein S16 (1:100, LS-C30572, Lifespan Bioscience), anti-SAP97 (1:1000, PA1-741, Affinity Bioreagents), anti-septin7 (1:100, 18991, IBL), anti-spectrin β (1:1000, MAB1622, Chemicon), and anti-α-tubulin (1:1000, T-9026, Sigma-Aldrich) antibodies were used in this study. Cy3- and horseradish peroxidase (HRP)-conjugated secondary antibodies were purchased from Jackson ImmunoResearch. Alexa488- and Alexa647-conjugated secondary antibodies were purchased from Life Technology.

### Cell Culture and Immunostaining

Cultured hippocampal neurons were prepared and maintained as described previously (Fiala et al., 1998; Furutani et al., 2007). Briefly, the hippocampus was dissected from embryonic days 16 mice and cultured in 35 mm-glass bottom dishes (P35G-0-10-C: Mattek or 3911-035-10: Asahi glass) coated with 0.2 mg/ml of poly-L-lysine hydrobromide (Nacalai tesque) at 5.6 × 10^4^ cells/dish. The neurons were cultured in minimum essential medium containing 5% FBS, 2% B27-supplement (Life Technology: 0080085SA), 0.5 mM glutamine, and penicillin/streptomycin. After 2.5 days, 10 μM cytosine β-D-arabinofuranoside (Ara-C) was added to the medium for the inhibition of glial cell growth. Cultured hippocampal neurons (14 DIV) were fixed with 4% PFA or 100% methanol for 10 min. After permeabilization with 0.25% Triton X-100 and blocking with 10% FBS, the neurons were incubated with primary antibodies or Alexa488-conjugated phalloidin (Life Technology) overnight at 4°C and visualized with Alexa Fluor or Cy dye-conjugated secondary antibodies. Single plane images or Z-stacked images (0.6 μm interval) were acquired with FV1000 confocal laser scanning microscopy (Olympus). The animal experiment was approved by RIKEN Institutional Animal Use and Care Administrative Advisory Committee.

### Purification of the Dendritic Phagocytic Cup Fraction

The hippocampus was dissected from wild-type (WT) and TLCN-deficient mice at embryonic day 16 and cultured on 35-mm plastic cell culture dishes (Corning; 430165) coated with 0.2 mg/ml of poly-L-lysine hydrobromide at 7 × 10^4^ cells/dish. Magnetic polystyrene microbeads (3 × 10^6^ particles/dish; 2.0–2.9 μm in diameter; PM-20-10; Sperotech) were added to 20 dishes containing the cultured neurons at 13 DIV. After 1 day, the neurons were washed with PBS 3 times and lyzed with 500 μl/dish of lysis buffer [PBS containing 0.01% Triton X-100, Complete EDTA free protease inhibitor cocktail (Roche), and PhosSTOP phosphatase inhibitor cocktail (Roche). The lysates were collected with a cell scraper and applied to silicone-coated microtubes, and then the magnetic beads were collected with a magnet apparatus. The supernatant was collected and used as an unbound fraction in silver staining and Western blot analysis. The beads collected in a silicone-coated microtube were washed 10 times using vortex mixer for 15 s each time with the lysis buffer. Proteins bound to the beads (bound fraction) were eluted by the addition of 50 μl of 1x SDS sample buffer (62.5 mM Tris HCl, pH 6.8, 2.5% SDS, and 10% glycerol) and boiling at 98°C for 5 min. Protein concentrations of the unbound and bound fractions were measured with BCA protein assay kit (Thermo Scientific).

### Silver Staining and Western Blot Analysis

The bound and unbound fractions (50 ng) were separated by SDS-PAGE, followed by silver staining (Silver staining kit II; Wako) or Western blotting.

### Mass Spectrometry Analysis

About 5 μg of bound fraction proteins prepared from 10 dishes (35 mm) of cultured hippocampal neurons were diluted in 1x SDS sample buffer containing 50 mM dithiothreitol, boiled at 98°C, separated in 5–20% SDS-polyacrylamide gel, fixed with 50% methanol and 7% acetic acid for 20 min, stained with SYPRO Ruby protein gel stain (Life technologies) overnight at room temperature, and washed with MilliQ water. The entire lane was divided into 24 gels and subjected to in-gel trypsin digestion according to the following procedure. The gels were further cut into small pieces and washed 3 times with 500 μl of MilliQ water for 10 min at 37°C. To remove SYPRO Ruby, the pieces were incubated with 100 μl of 50 mM NH_4_HCO_3_ and 50% CH_3_CN for 10 min at 37°C. The destained pieces were dehydrated with 50 μl of CH_3_CN for 10 min at 37°C and dried in a vacuum centrifuge. The pieces were reduced with 50 μl of 10 mM dithiothreitol in 100 mM NH_4_HCO_3_ for 15 min at 50°C and alkylated with 2 μl of 250 mM iodoacetamide in 100 mM NH_4_HCO_3_ for 15 min at room temperature. The pieces were washed with 50 μl of 100 mM NH_4_HCO_3_, 50 μl of 50 mM NH_4_HCO_3_ in 50% CH_3_CN, and dried in a vacuum centrifuge. The dried pieces were immersed with 20 μl of 10 ng/μl modified trypsin (Promega) in 50 mM acetic acid overnight at 37°C. The trypsin-digested peptides were extracted from the pieces by the incubation with 50 μl of 50% CH_3_CN and 1% TFA for 10 min at 37°C, 50 μl of 25% CH_3_CN, 20% HCOOH, 15% isopropanol in MilliQ water for 15 min at 37°C, and 50 μl of 80% CH_3_CN for 2 min at 37°C. The extracts were mixed and dried in a vacuum centrifuge. The resulting peptides from individual pieces were dissolved into 2% CH_3_CN and 0.1% TFA. Each of the samples was loaded onto a C18 reverse-phase capillary column (L-column2 ODS, 0.1 × 150 mm, particle size; 3 μm, Chemicals Evaluation and Research Institute). The peptides were separated with a liner gradient (30 min, 5–65% CH_3_CN/0.1% HCOOH) at a flow rate of 0.5 μl/min. Eluted peptides were ionized under 1.8 kV of ion spray voltage and detected in a scanned mass range from 400 and 2000 m/z on an LTQ linear ion trap mass spectrometer (Thermo Fisher Scientific).

### Data Analysis

Protein identification from the resulting MS and MS/MS data was performed by searching the mouse protein subset of the NCBI non-redundant protein database using Mascot software (Matrix Science). For protein identification by Mascot, quantified peptides with a mascot ion score ≥ 15 were used. We used the NCBI non-redundant multiple protein database for description of proteins that have several names and IDs. To integrate several protein names and IDs, Ingenuity Pathway Analysis software (Ingenuity systems) was used. Proteomic analysis experiments were performed 3 times with WT hippocampal neurons and once with TLCN-deficient hippocampal neurons. To remove non-specifically bound proteins, we selected the proteins that were reproducibly detected in three independent experiments with WT neurons and that were not observed from TLCN-deficient neurons.

To compare the amount of protein in the dendritic phagocytic cup fraction, an abundance index was calculated (Peng et al., 2004). An abundance index of each protein was derived based on the number of peptides identified for each protein. It was calculated by the formula: (the total number of peptides identified/molecular weight) × 50,000, assuming that the average mass of proteins is 50 kDa.

### GO Term and Pathway Analysis

To examine whether particular proteins were enriched in the dendritic phagocytic cup fraction, DAVID Web tool was used for GO terms analysis (Huang et al., 2009). It was performed against DAVID’s GO biological process FAT category and only GO terms with a *P*-value < 1 × 10^-3^ were considered enriched.

## Results

### Morphological and Molecular Resemblance Between Dendritic Filopodia and Phagocytic Cups

**FIGURE 1:**
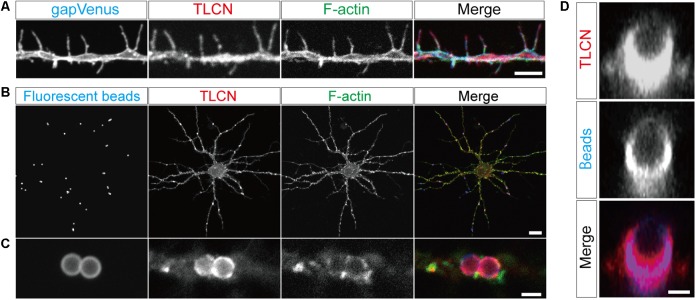
Localization of TLCN in dendritic filopodia and phagocytic cups. **(A)** Immunofluorescence staining of a dendrite of a cultured hippocampal neuron expressing gapVenus with anti-GFP antibody (blue in a merged image), anti-TLCN antibody (red in a merged image), and phalloidin (green in a merged image). TLCN and F-actin are abundantly observed in dendritic filopodia. **(B,C)** Formation of phagocytic cup structures on neuronal dendrites. Fluorescent microbeads (blue in merged images of **B,C**) added to cultured hippocampal neurons strongly adhere onto dendrites and induce the accumulation of TLCN (red in merged images of **B,C**) and F-actin (green in merged images of **B,C**). **(D)** A lateral view of a dendritic phagocytic cup reconstructed from confocal images reveals surrounding of the fluorescent bead (blue in merged images of **D**) by TLCN (red in merged images of **D**). Scale bars, 1 μm in **(D)**, 2 μm in **(A)** and **(C)**, 20 μm in **(B)**.

The cell adhesion molecule, TLCN, is highly present in dendritic filopodia and shafts (**Figure 1A**) and regulates dendritic morphology through the interaction with its extracellular matrix ligand, VN, and its intracellular binding partners, ERM proteins (Matsuno et al., 2006; Furutani et al., 2007, 2012). Interestingly, when polystyrene microbeads are put into culture medium of hippocampal neurons, they immediately adsorb VN, an extremely adhesive protein abundantly present in the serum, and then bind onto neuronal dendrites to induce unique membranous protrusions, phagocytic cups, in a TLCN-dependent manner (Esselens et al., 2004; Furutani et al., 2012) (**Figures 1B,C**). In the phagocytic cups, dendritic plasma membranes protrude from dendritic shafts, almost covering the lateral surface of the microbeads (**Figure 1D**). Notably, intracellular signaling molecules downstream of TLCN cascade in dendritic filopodia accumulate also in phagocytic cups, including F-actin (**Figure 1C**), phosphorylated ERM, and PI(4,5)P_2_ (Furutani et al., 2012). Thus, both dendritic filopodia and phagocytic cups are membranous protruding structures on neuronal dendrites and they significantly share functional molecular constituents. We hence reasoned that the TLCN-accumulating phagocytic cups on dendrites can serve as a substitute for dendritic filopodia and performed the following purification and proteomics analyses.

### Purification of Proteins Enriched in Dendritic Phagocytic Cups

**FIGURE 2:**
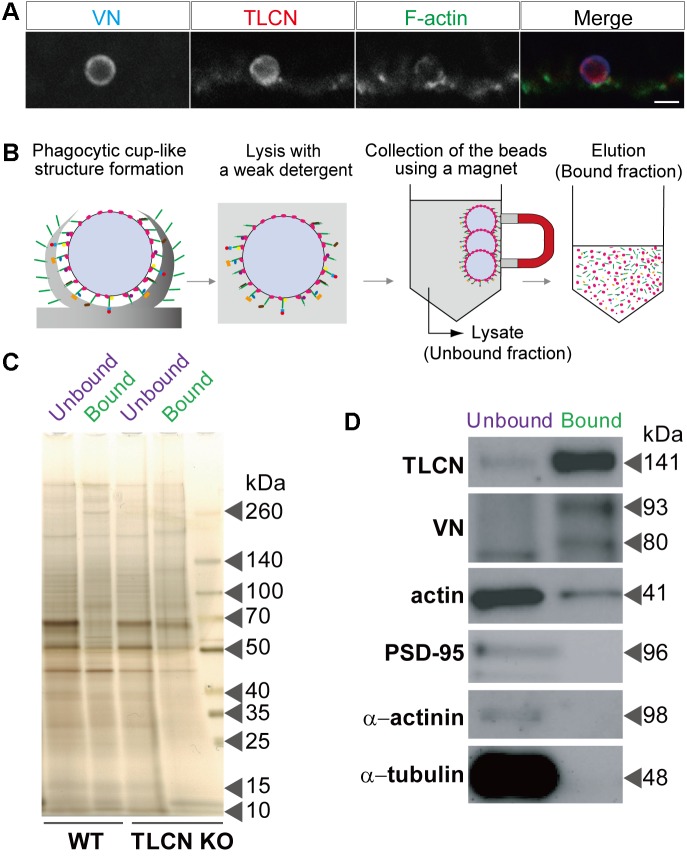
Purification of proteins enriched in dendritic phagocytic cups. **(A)** A dendritic phagocytic cup induced by a magnetic microbead attached onto a neuronal dendrite and immunostained with anti-VN antibody (blue in merged images of **A**), anti-TLCN antibody (red in merged images of **A**), and phalloidin (green in merged images of **A**). Scale bar, 2 μm in **(A)**. **(B)** A schematic diagram illustrating the purification procedure of dendritic phagocytic cup fraction. Magnetic microbeads were added onto cultured hippocampal neurons to induce the formation of dendritic phagocytic cups. After 1 day of incubation, neurons was solubilized with lysis buffer containing 0.01% Triton X-100. The beads were separated from unbound fraction using a magnet. After washing, bound proteins were eluted with SDS-containing buffer. Red: VN, green: TLCN, other colors: bound proteins. **(C)** Silver staining of proteins in the microbeads-unbound and bound fractions. Same amount (50 ng) of proteins in the unbound and bound fractions purified from wild-type (WT) and TLCN-deficient (TLCN KO) hippocampal neurons were separated by SDS-PAGE and visualized with silver staining. **(D)** Western blot analysis of the unbound and bound fractions. Same amount (50 ng) of proteins were separated by SDS-PAGE and subjected to Western blot analysis using anti-TLCN, anti-VN, anti-actin, anti-PSD-95, anti-α-actinin, and anti-α-tubulin antibodies. Molecular weights of individual proteins were estimated from molecular weight markers and shown on the right. Note that TLCN, VN (arrow heads), and actin are observed in the dendritic phagocytic cup fraction.

By taking advantage of the specific and strong binding of VN-coated beads onto TLCN localized to neuronal dendrites, we devised a unique method for purification of proteins enriched in dendritic phagocytic cups. Similar to polystyrene microbeads, the addition of magnetic microbeads to cultured hippocampal neurons efficiently induced the formation of phagocytic cups on dendrites (**Figure 2A**). The neurons with those phagocytic cups were solubilized with lysis buffer containing mild detergent (0.01% Triton X-100) and then the magnetic beads were collected using a magnet. The proteins bound to the microbeads were eluted with 2.5% SDS-containing solution (**Figure 2B**). Silver staining of protein constituents following SDS-PAGE could not reveal any marked differences between the microbeads-bound and -unbound fractions prepared from both WT and TLCN-deficient hippocampal neurons (**Figure 2C**). However, Western blot analysis validated the high abundance of TLCN and VN, as well as the significant presence of actin, in the microbeads-bound fraction (**Figure 2D**). In contrast, PSD-95, α-actinin, and β-tubulin were not detected in the microbeads-bound fraction (**Figure 2D** and **Supplementary Figure S1**). Thus, the proteins associated with TLCN in the dendritic phagocytic cups were efficiently concentrated in the microbeads-bound fraction, which was next subjected to a comprehensive proteomic analysis.

### Proteomics Analysis of Dendritic Phagocytic Cups

**FIGURE 3:**
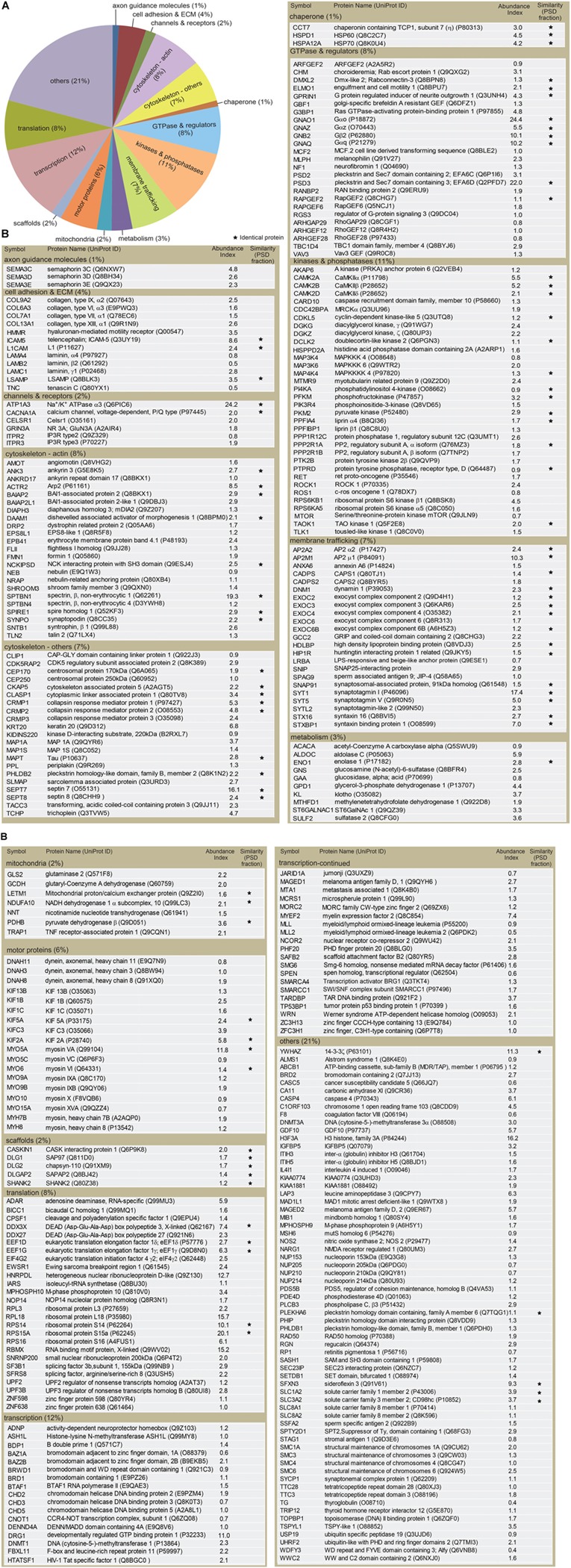
Classification of proteins identified in the dendritic phagocytic cups. **(A)** Pie chart showing functional categories of the proteins in the dendritic phagocytic cups. Axon guidance (1%), cell adhesion and ECM (4%), channels and receptors (2%), cytoskeleton-actin (8%), cytoskeleton-others (7%), chaperone (1%), GTPase and regulators (8%), kinase and phosphatase (11%), membrane trafficking (7%), metabolism (3%), mitochondria (2%), scaffolds (2%), transcription (12%), translation (8%), and others (21%) are shown. **(B)** Protein components in the dendritic phatocytic cup fraction. Symbol, protein name, UniProt ID, abundance index, and similarity with the 984 proteins in mouse PSD fraction described by Bayes et al. (2012) are indicated in the table. An abundance index for each protein was calculated from number of fragments detected by LC-MS/MS and normalized with molecular weight. The proteins identified in both dendritic phagocytic cup and PSD fractions are marked with asterisks.

To uncover molecular constituents in the dendritic filopodia, proteins in the purified phagocytic cup fraction were separated by SDS-PAGE, stained with SYPRO Ruby, divided into 24 gel pieces, and then trypsinized. The resulting peptide fragments were analyzed by liquid chromatography-tandem mass spectroscopy (LC-MS/MS). As a negative control, we used cultured hippocampal neurons prepared from TLCN-deficient mice, onto which the magnetic microbeads non-specifically and weakly bound without forming any phagocytic cups. As a result, 731 proteins were reproducibly observed in three independent experiments from WT neurons, while 412 proteins among them were detected also from TLCN-deficient mice (**Supplementary Data Sheet S1**). Thus, the subtracted 319 molecules were identified as proteins enriched in the TLCN-containing phagocytic cups (**Figure 3**).

The identified proteins were classified into the following functional categories: axon guidance molecules (1%), cell adhesion and ECM (4%), channels and receptors (2%), cytoskeleton-actin (8%), cytoskeleton-others (7%), chaperone (1%), GTPase and regulators (8%), kinases and phosphatases (11%), membrane trafficking (7%), metabolism (3%), mitochondria (2%), scaffolds (2%), transcription (12%), translation (8%), and others (21%) (**Figure 3A**). Abundance index for each protein was calculated from the number of peptide fragments detected by LC-MS/MS and normalized with its molecular weight. Eighty-four proteins were commonly present in both dendritic phagocytic cups and PSD fractions (Bayes et al., 2012) (**Figure 3B**, asterisks). The 319 proteins were sorted according to their abundance indices, and the top 40 proteins are shown in **Table 1**. Gαo, Na^+^/K^+^ ATPase α3, and EFA6D were most abundantly present in the fraction. TLCN was ranked at 20th with abundance index of 8.6, demonstrating the successful purification of proteins contained in TLCN-containing phagocytic cups.

**Table 1:** Top 40 proteins abundantly present in the dendritic phagocytic cup fraction.

Rank	Symbol	Entrez gene name	Abundance index	Functional categories	Dendritic filopodia localization
1	GNAO1	Gαo	24.4	GTPase and regulators	++
2	ATP1A3	Na^+^/K^+^ ATPase α3	24.2	Transportor	++
3	PSD3	Pleckstrin and Sec7 domain containing 3 (EFA6D)	22.0	GTPase and regulators	++
4	RPS15A	Ribosomal protein S15a	20.1	Translation	+
5	SPTBN1	Spectrin, β, non-erythrocytic 1	19.3	Cytoskeleton actin	++
6	SYT1	Synaptotagmin I	17.4	Membrane trafficking	–
7	H3F3A	H3 histone, family 3A	16.2	Others	–
8	SEPT7	Septin 7	16.1	Cytoskeleton others	++
9	RPL18	Ribosomal protein L18	15.7	Translation	+
10	RBMX	RNA binding motif protein, X-linked (hnRNP G)	15.2	Translation	N.D.
11	HNRPDL	Heterogeneous nuclear ribonucleoprotein D-like	12.7	Transcription	N.D.
12	MYO5A	Myosin VA	11.8	Motor proteins	++
13	YWHAZ	14-3-3ζ	11.3	Others	–
14	DRG1	Developmentally regulated GTP binding protein 1	11.0	Transcription	N.D.
15	AP2M1	Adaptor-related protein complex 2, μ1 subunit (AP2μ1)	10.3	Membrane trafficking	N.D.
16	GNAQ	Gαq	10.2	GTPase and regulators	++
17	RPS14	Ribosomal protein S14	10.1	Translation	+
18	GNB2	Gβ2	10.1	GTPase and regulators	++
19	SFXN3	Sideroflexin 3	9.3	Transporter	N.D.
20	ICAM5	Intercellular adhesion molecule 5, telencephalin	8.6	Cell adhesion	++
21	ACTR2	Arp2	8.5	Cytoskeleton actin	++
22	MYEF2	Myelin expression factor 2	7.4	Transcription	N.D.
23	DDX3X	DEAD (Asp-Glu-Ala-Asp) box polypeptide 3, X-linked	7.4	Others	N.D.
24	STXBP1	Syntaxin binding protein 1	7.0	Membrane trafficking	–
25	KRT20	Keratin 20	6.8	Cytoskeleton others	–
26	EEF1G	Eukaryotic translation elongation factor 1 γ (eEF1γ)	6.3	Translation	++
27	LAP3	Leucine aminopeptidase 3	6.3	Protease	N.D.
28	CASP4	Caspase 4, apoptosis-related cysteine peptidase	6.1	Protease	N.D.
29	RPS16	Ribosomal protein S16	6.1	translation	++
30	ADAR	Adenosine deaminase, RNA-specific	5.9	Translation	–
31	ALDOC	Aldolase C, fructose-bisphosphate	5.9	Others	–
32	KIF2A	Kinesin heavy chain member 2A	5.8	Motor proteins	–
33	MAGED2	Melanoma antigen family D, 2	5.7	Others	N.D.
34	GDF10	Growth differentiation factor 10	5.7	Extracellular	N.D.
35	GNAZ	Gαz	5.5	GTPase and regulators	N.D.
36	CAMK2A	CaMKIIα	5.5	Kinase	++
37	CRMP1	Collapsin response mediator protein 1	5.3	Others	N.D.
38	CAMK2B	CaMKIIβ	5.2	Kinase	++
39	SYT5	Synaptotagmin V	5.0	Membrane trafficking	–
40	G3BP1	GTPase activating protein (SH3 domain) binding protein 1	4.8	GTPase and regulators	N.D.


*The identified 319 proteins are sorted by abundance index, and then top 40 proteins are shown. TLCN is ranked at 20th. Proteins localized to dendritic filopodia in this study are marked by (++). Arp2 and CaMKIIβ are localized to dendritic filopodia in previous studies (Shen et al., 1998; Hotulainen and Hoogenraad, 2010) and also marked by (++). Proteins that make complex with ribosomal proteins and possibly localized to dendritic filopodia are marked by (+). Proteins that are not localized to dendritic filopodia and not determined the localization are marked by (–) and N.D., respectively.*

To find out biological meanings behind the list of proteins enriched in phagocytic cups, we used DAVID functional annotation software that can identify over-represented Gene Ontology (GO) terms (Huang et al., 2009). This analysis revealed several important cellular pathways including cytoskeletal organization, exocytosis, secretion, actin filament-based process, microtubule-based process, small GTPase regulation, and neuronal development (**Table 2**), all of which are closely related to structural and functional properties of both dendritic filopodia and phagocytic cups.

**Table 2:** Gene ontology (GO) terms associated with the dendritic phagocytic cup fraction.

GO identifier	Term	Count	Fold enrichment	*P*-value	Benjamini
0007010	Cytoskeleton organization	22	3.7	4.96E-07	7.14E-04
0006887	Exocytosis	12	6.0	4.82E-06	0.002
0030029	Actin filament-based process	14	4.4	1.90E-05	0.007
0007017	Microtubule-based process	15	3.9	3.04E-05	0.009
0032940	Secretion by cell	14	4.1	3.41E-05	0.008
0030030	Cell projection organization	18	3.1	7.18E-05	0.011
0046903	Secretion	14	3.5	1.96E-04	0.028
0030036	Actin cytoskeleton organization	12	4.0	2.08E-04	0.027
0051056	Regulation of small GTPase mediated signal transduction	14	3.4	2.67E-04	0.031
0051493	Regulation of cytoskeleton organization	9	5.0	4.27E-04	0.046
0046578	Regulation of Ras protein signal transduction	12	3.6	4.60E-04	0.046
0007018	Microtubule-based movement	9	4.9	4.89E-04	0.046
0033043	Regulation of organelle organization	11	3.9	4.95E-04	0.044
0044275	Cellular carbohydrate catabolic process	7	6.4	7.32E-04	0.060
0048666	Neuron development	15	2.8	8.90E-04	0.069
0051495	Positive regulation of cytoskeleton organization	5	10.6	0.001	0.083


*The table shows GO terms that are enriched in the dendritic filopodia fraction and identified by DAVID functional annotation software. The top ranked GO terms (*p* < 0.001) are selected from Biological Process of GO terms. Count is number of cluster proteins annotated with a given GO term. Fold Enrichment shows enrichment of cluster proteins based on proteins present in mouse genome. *P*-values are adjusted by Benjamini–Hochberg procedure in DAVID.*

### Localization of Identified Proteins in Dendritic Filopodia and Phagocytic Cups

**FIGURE 4:**
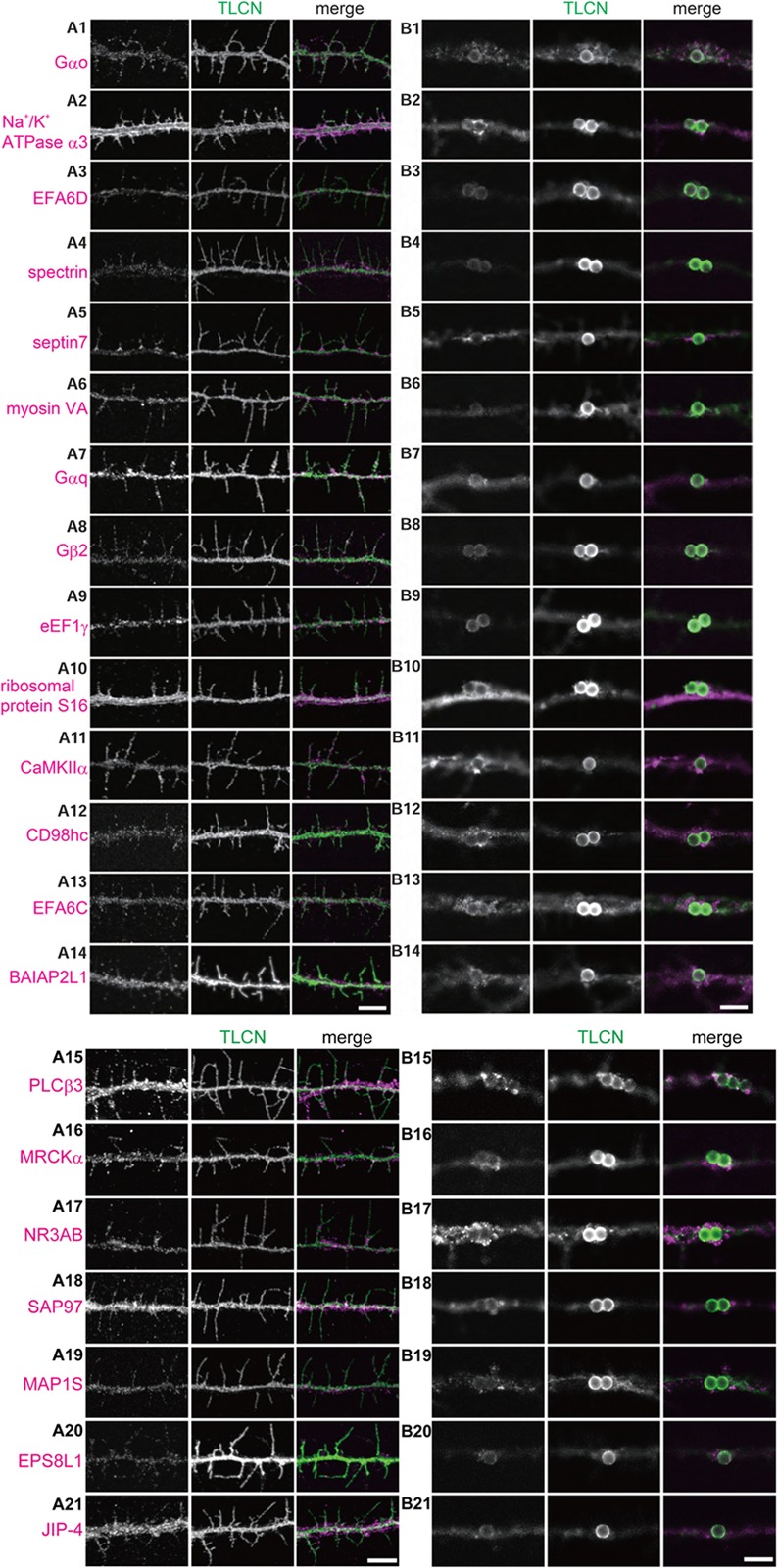
Validation of the identified proteins in dendritic filopodia. **(A,B)** Localization of the identified proteins in dendritic filopodia **(A1–A21)** and phagocytic cups **(B1–B21)** at 14 DIV hippocampal neurons. The cultured neurons were immunostained with anti-TLCN antibody and specific antibodies against Gαo **(A1,B1)**, Na^+^/K^+^ ATPase α3 **(A2,B2)**, EFA6D **(A3,B3**), spectrin **(A4,B4)**, septin7 **(A5,B5)**, myosin VA **(A6,B6)**, Gαq **(A7,B7)**, Gβ2 **(A8,B8)**, eEF1γ **(A9,B9)**, ribosomal protein S16 **(A10,B10)**, CaMKIIα **(A11,B11)**, CD98hc **(A12,B12)**, EFA6C **(A13,B13)**, BAIAP2L1 **(A14,B14)**, PLCβ3 **(A15,B15)**, MRCKα **(A16,B16)**, NR3A/B (GluN3A/B) **(A17,B17)**, SAP97 (SLC3A2) **(A18,B18)**, MAP1S **(A19,B19)**, EPS8L1 **(A20,B20)**, and JIP-4 (SPAG9) **(A21,B21)**. In merged images, the identified proteins and TLCN are shown in magenta and green, respectively. Single plane of images focused on dendritic filopodia **(A1–A21)** and center of microbeads **(B1–B21)** were acquired using a confocal microscopy. Scale bars, 5 μm in **(A14,A21,B14,B21)**.

We next asked whether the proteins identified by the proteomics analysis are actually present in dendritic filopodia and phagocytic cups by immunostaining of cultured hippocampal neurons with specific antibodies. Among 46 proteins examined, 21 proteins were abundantly present in dendritic filopodia as well as in phagocytic cups (**Figures 4A,B**). Eleven proteins were localized to axon, dendritic shaft, and cell body. Localizations of the remaining 14 proteins could not be determined because of poor quality of antibodies. Many of the proteins showed unique localization patterns in both dendritic filopodia and phagocytic cups. For example, GTP-binding proteins and downstream effector enzymes such as Gαo, Gαq, Gβ2, CaMKIIα, and PLCβ3 were mostly found in punctates along dendritic filopodia (**Figures 4A1,A7,A8,A11,A15**), indicating the presence of “hot spots” for intracellular signaling cascades. Different cytoskeletal proteins displayed distinct localizations along the proximo-distal axis of dendritic filopodia: myosin VA in the distal region (**Figure 4A6**), spectrin in the proximal region (**Figure 4A4**), and septin 7 at the filopodial base (**Figure 4A5**). Also in phagocytic cups, septin 7 showed a characteristic pattern of localization at the interface between microbeads and dendritic shaft (**Figure 4B5**). Molecules involved in phagocytosis were strongly accumulated in phagocytic cups as well as dendritic filopodia, including MRCKα, and EPS8L1 (**Figures 4A,B16,B20**). Unexpectedly, ribosomal protein S16 and elongation factor eEF1γ were abundantly present in dendritic filopodia (**Figures 4A9,A10**). Together with the fact that other ribosomal subunits S14, S15a, and L18 were contained in the top 40 list, it is conceivable that protein translation machinery exists in dendritic filopodia, as was demonstrated in dendritic spines. Thus, many of the identified proteins were verified to be present in dendritic filopodia and phagocytic cups and localized to distinct domains possibly for their proper functioning.

## Discussion

Despite multiple lines of evidence for the structural and functional significance of dendritic filopodia as the precursor of spines, it has been largely unknown what functional molecules are contained in the dendritic filopodia. This is because there is no effective method to selectively collect dendritic filopodia-enriched fraction from neurons. Instead, we made use of the specific and strong binding between the dendritic filopodia adhesion molecule TLCN and its extracellular ligand VN. TLCN is a key regulator for dendrite morphogenesis, playing a pivotal role in dendritic filopodia formation and maintenance as well as filopodia-to-spine transition, together with its extracellular ligand VN (Matsuno et al., 2006; Furutani et al., 2007, 2012). VN-coated microbeads attached onto TLCN on neuronal dendrites and induced unique membrane protrusions called dendritic phagocytic cups, which was reminiscent of dendritic filopodia in respect with their protruding morphology and shared molecular constituents such as TLCN and phosphorylated ERM proteins (Furutani et al., 2012). In the present study, we succeeded in magnetically collecting proteins enriched in dendritic phagocytic cups on cultured hippocampal neurons and profiled the 319 proteins contained in it. Immunocytochemical analysis revealed that about half of the identified proteins are actually present in dendritic filopodia as well as in dendritic phagocytic cups. Thus, to the best of our knowledge, this is the first report describing the proteomics profile of dendritic filopodia.

We compared the 319 proteins in dendritic phagocytic cups identified in this study with the 984 proteins in mouse postsynaptic density fractions previously described by Bayes et al. (2012). Although 84 proteins (26%) were observed in both dendritic spines and phagocytic cups, a larger number of proteins (74%) were detected specifically to the dendritic phagocytic cups or filopodia. Thus, it is obvious that the protein profile in the dendritic filopodia is remarkably different from that in the spines.

The dendritic spines are equipped with the translation machinery for local protein synthesis that is important for synaptic plasticity. The present proteome of dendritic filopodia also contains several molecules involved in protein translation, such as ribosomal protein subunits (L3, L18, S14, S15a, S16) and initiation/elongation factors (eIF4γ2, eEF1δ, eEF1γ). We have confirmed that some of these molecules ribosomal protein S16, eEF1γ) are actually present in the dendritic filopodia. These results suggest that the local protein synthesis may occur also in the dendritic filopodia similar to the spines.

One of the most conspicuous differences in protein constituents between the dendritic filopodia and spines is their repertoires of actin-binding molecules. Although both dendritic filopodia and spines are actin-rich protrusions, the structural modes of actin polymerization are different: unbranched, straight actin filaments in dendritic filopodia vs. mesh-like, highly branched actin filaments in spines. Interestingly, our analysis revealed that the dendritic phagocytic cups and filopodia contain several actin-binding proteins such as mDia2, DAAM1, formin 1, flightless 1 homolog, and spire homolog 1, all of which mediate the formation of unbranched, straight actin filaments (Campellone and Welch, 2010). In contrast, dendritic spines contain multiple subunits of Arp2/3 and cofilin1 that play crucial roles in polymerization and stabilization of branched filamentous actin (Bayes et al., 2012). Thus, the results of proteomic analyses faithfully reflect distinct morphology of actin filaments in dendritic protrusions.

Another clear difference between the filopodia and spines lies in their compositions of receptors and scaffold proteins. In the spines, the presence of 20 receptors and 25 scaffold proteins were reported (Peng et al., 2004). By contrast, only 2 receptors (NR3A, Celsr1) and 5 scaffold proteins (CASK-interacting protein 1, SAP97, PSD-93, SAPAP2, Shank 2) were detected in our proteomics analysis. These results are consistent with the notion that most of the synaptic receptors and scaffold proteins are incorporated into dendritic protrusions at relatively late stages of development. Noteworthy is the presence of a unique NMDA receptor subunit, NR3A, in the dendritic filopodia, whose ontogenic expression peaks during early postnatal period in parallel with dendrite morphogenesis (Ito, 2002). In addition, we identified an atypical microtubule-associated protein, MAP1S, which was reported to interact with NR3A and to be present in β-tubulin III-negative filopodia-like protrusions in dendrites (Kayser et al., 2008). Interestingly, both NR3A- and TLCN-knockout mice display accelerated synapse maturation and enlarged spine heads (Matsuno et al., 2006; Kitanishi et al., 2010), suggesting that these two molecules in the dendritic filopodia may serve as physiological and morphological brakes of synaptogenesis, respectively (Ito, 2002; Matsuno et al., 2006).

In summary, our comprehensive analysis of dendritic filopodia will provide a useful resource for neuroscientists studying neural development and plasticity at molecular and cellular levels.

## Ethics Statement

This study was carried out in accordance with the recommendations of RIKEN Institutional Animal experiment guideline, RIKEN Institutional Animal Use and Care Administrative Advisory Committee. The protocol was approved by RIKEN Institutional Animal Use and Care Administrative Advisory Committee.

## Author Contributions

YF and YY: studied the conception, designed, and drafted the manuscript. YF: acquisition of data, analysis and interpretation of the data.

## Conflict of Interest Statement

The authors declare that the research was conducted in the absence of any commercial or financial relationships that could be construed as a potential conflict of interest.

## Supplementary Material

**FIGURE S1 |** Original images for Western blot analysis of the unbound and bound fractions. Proteins (50 ng) from unbound and bound fractions were separated by SDS-PAGE and subjected to Western blot analysis with anti-TLCN, anti-actin, anti-VN, anti-PSD-95, anti-α-actinin, and anti-α-tubulin antibodies. Positions of molecular weight markers are shown on the left.

**DATA SHEET S1 |** Proteins detected in the dendritic phagocytic cup fraction. Symbol, gene name, abundance index, average of abundance index, and standard deviation (SD) are indicated in the table. The dendritic phagocytic cup fractions were purified three times from wild-type and once from TLCN-deficient (KO) hippocampal neurons. Each abundance index was calculated and shown as abundance 1, 2, 3, and KO, respectively. In three independent experiments, 731 proteins were reproducibly identified, while 412 proteins were also detected from TLCN-deficient neurons. Thus, 319 proteins were regarded as TLCN-dependent and dendritic phagocytic cup-enriched molecules, whereas the rest (412 proteins) were TLCN-independent, non-specific ones. n.d. means “not detected” and error indicates SD.

## ABBREVIATIONS

DIV: days *in vitro*
ERM: ezrin/radixin/moesin; TLCN, Telencephalin
VN: vitronectin
