# Supplementary figures and images for: Proteomic Analysis of Dendritic Filopodia-Rich Fraction Isolated by Telencephalin and Vitronectin Interaction
Source: Front Synaptic Neurosci. 2018 Aug 10;10:27. doi: 10.3389/fnsyn.2018.00027 (PMC6097459; doi:10.3389/fnsyn.2018.00027)

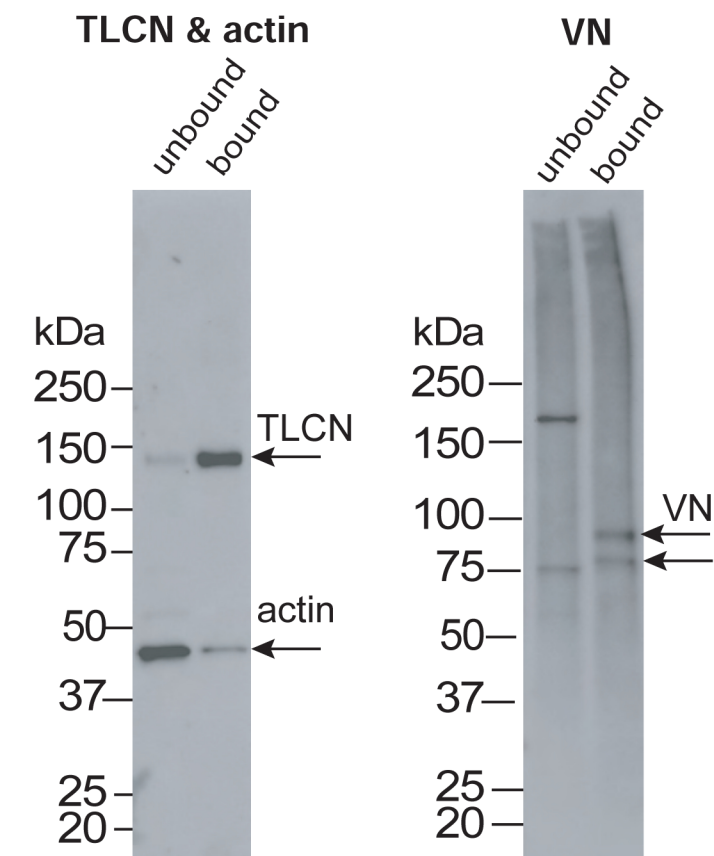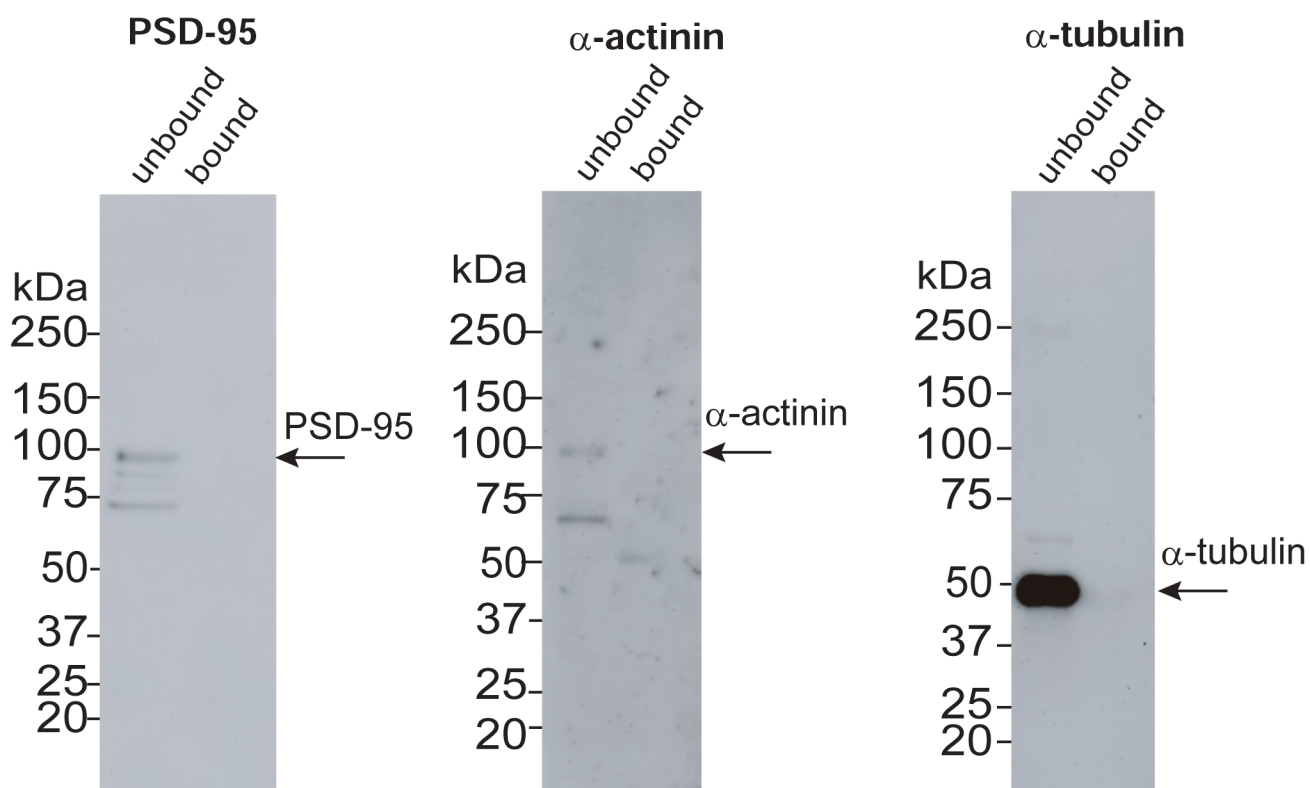

Supplement: Supplementary file 1 [file Image_1.pdf]
